# Redox-Active Gel Electrolyte Combined with Branched Polyaniline Nanofibers Doped with Ferrous Ions for Ultra-High-Performance Flexible Supercapacitors

**DOI:** 10.3390/polym11081357

**Published:** 2019-08-16

**Authors:** Youtian Mo, Wei Meng, Yanlin Xia, Xusheng Du

**Affiliations:** 1 Institute of Advanced Wear & Corrosion Resistance and Functional Materials, Jinan University, Guangzhou 510632, China; 2 School of Aerospace, Mechanical and Mechatronic Engineering J07, University of Sydney, Sydney, NSW 2006, Australia

**Keywords:** polyaniline, branched nanofibers, redox-active gel electrolyte, flexible supercapacitor

## Abstract

In this work, the effects of utilizing an Fe^2+^/Fe^3+^ redox-active electrolyte and Fe^2+^-doped polyaniline (PANI) electrode material on the performance of an assembled supercapacitor (SC) were studied. The concentration of the redox couple additive in the electrolyte of the SC was optimized to be 0.5 M. With the optimized concentration of 0.4 M Fe^2+^, the doped PANI branched nanofibers electropolymerized onto titanium mesh were much thinner, cleaner, and more branched than normal PANI. A specific capacitance (C_s_) of 8468 F g^−1^ for the 0.4 M Fe^2+^/PANI electrode in the 1 M H_2_SO_4_ + 0.5 M Fe^2+^/Fe^3+^ gel electrolyte and an energy density of 218.1 Wh kg^−1^ at a power density of 1854.4 W kg^−1^ for the resultant SC were achieved, which were much higher than those of the conventional PANI electrode tested in a normal H_2_SO_4_ electrolyte (404 F g^−1^ and 24.9 Wh kg^−1^). These results are among the highest reported for PANI-based SCs in the literature so far and demonstrate the potential effectiveness of this strategy to improve the electrochemical performance of flexible SCs by modifying both the electrode and electrolyte.

## 1. Introduction

Supercapacitors (SCs) are attractive because of their high power, long periodicity, and environmental friendliness [1,2]. The electrode materials used in SCs include carbon-based materials, conductive polymers, and transition metal oxides/hydroxide [2,3,4,5,6]. Polyaniline (PANI) is not only easy to prepare but also has good conductivity, excellent electrochemical activity, fast Faraday reactivity, facile fabrication, and so forth. [7]. Nonetheless, long-term charge and discharge easily cause volume expansion and contraction, which leads to a decrease in mechanical and electrochemical performance, thereby limiting its application in SCs [8]. So far, some strategies have been developed to promote its electrochemical behaviors, including combinations with other materials, such as PANI/carbon-based materials [9,10], PANI/metallic oxide [11,12], and PANI/metallic oxides/carbon materials [13], or the doping of PANI with certain materials/ions, including protonic acids/anions (HCl [14], H_2_SO_4_ [15], HClO_4_ [16], and H_3_PO_4_ [17]) and metallic cations (LiCl [18], Ni^2+^ [19], Co^2+^ [20], Fe^3+^ [21], Zn^2+^ [22,23], Cu^2+^ [24], and Mn^2+^ [25]).

The effective doping of electrodes could produce electroactive materials with certain physicochemical structures and provide enough and suitable active sites which would favor the electrochemical process in devices [26,27,28]. A common, well-investigated method is the anion/protonic acid doping of PANI. PANI nanofibers, nanorods, nanoparticles, and pyramid-like PANI have been deposited uniformly on carbonized kapok surfaces by using HCl, H_2_SO_4_, HClO_4_, HPO_4_, and PTSA as dopants, and their specific capacitances (C_s_) were measured to be 241, 245, 580, 233, and 289 F g^−1^ at 1.0 A g^−1^, respectively [17]. Recently, transition metal ions and other cations have been used as dopants for PANI due to their unique electronic exchange properties [20]. Doping cations in PANI can not only be used as redox catalysts and corrosion inhibitors [29,30] but also to enhance the energy storage of the polymer in certain cases. PANI/Co^2+^, PANI/Ni^2+^, PANI/Cu^2+^, PANI/Zn^2+^, and PANI/Fe^3+^ films have been prepared by electrodeposition and they displayed a higher specific capacitance than that of pure PANI [19,20,21,22,23]. It was found that PANI/Mn^2+^ film has more reaction active centers than pure PANI film due to the existence of metal ion dopants [21]. Moreover, compared with pure PANI, the addition of Co^2+^ to PANI is believed to widen the localization of the charge on the macromolecular skeleton due to the transformation of the quinoid ring structures to benzenoid rings by protonation [20,31].

To improve the performance of SCs (e.g., their energy density), two approaches have been adopted. One is developing new electrode materials and their composites [32,33]. Another is achieved by adjusting the configuration of the devices or the design and utilization of specific electrolytes [34]. Generally, novel electrode materials and their assembled symmetric/asymmetric SCs with high electrochemical performance can be designed and fabricated, including the above-described doped PANI electrode materials. For the utilization of specific electrolytes, besides organic electrolytes (such as ionic liquids) with a widening working potential window, redox-active electrolytes were recently developed by adding redox additives to electrolytes [35]. These redox media can afford additional capacitive contributions through their reversible electrochemical reactions and can improve the electrochemical performance of assembled symmetric/asymmetric SCs. Electrolytes with a variety of redox-active additions have been studied, such as H_2_SO_4_ + FeBr_3_ and KCl + VOSO_4_ [36,37], H_2_SO_4_ + KI, H_2_SO_4_ + VO^2+^/VO_2_^+^ and KOH + K_3_Fe(CN)_6_ [38,39,40], H_2_SO_4_ + hydroquinone [41], and H_2_SO_4_ + Fe^2+^/Fe^3+^ [42,43,44]. Among these, active electrolytes containing Fe^3+^/Fe^2+^ redox couples are easily available and cost effective, and they have been shown to be promising electrolytes in SCs, where PANI has been found to exhibit an enhanced C_s_ of 1062 F g^−1^ at a current density of 2 A g^−1^ [42].

It is expected that advanced SCs with enhanced capacitive performance will be developed by combining the advances in both electrodes and the electrolyte. Although various polyaniline material electrodes and redox-active electrolytes have been used in capacitors, little information on the capacitive performance of Fe^2+^-doped PANI in an Fe^3+^/Fe^2+^ active electrolyte is available so far. In this work, flexible symmetric SCs were designed and fabricated by both PANI electrode materials doped with Fe^2+^ and utilizing a redox-active electrolyte containing an Fe^2+^/Fe^3+^ additive. The effects of the presence of Fe^2+^ on the physicochemical structure and performance of the modified PANI electrode materials were investigated and the electrode materials were optimized. Moreover, the influence of incorporating an Fe^2+^/Fe^3+^ redox-active additive into the electrolyte combined with the optimized electrode materials on the performance of SCs was studied in detail.

## 2. Experimental

### 2.1. Preparation of PANI/Fe^2+^ and PANI Materials

PANI materials were electrodeposited in a three-electrode system on a CHI 760e electrochemical work station (CH Instruments, Inc., Austin, TX, USA). A saturated calomel electrode (SCE), a platinum net, and titanium mesh with a working area of 1 cm^2^ were used as the reference electrode, the counter electrode, and the working electrode, respectively. Before depositing the polymer, the titanium mesh was washed in an ultrasonic bath of ethanol for several minutes and finally air-dried at 70 °C. The electropolymerization of PANI was prepared by conducting a cyclic voltammetry (CV) test in a mixture of 0.5 M HCl and 0.2 M aniline with 0.2, 0.4, and 0.8 M FeCl_2_ in solution at a scanning rate of 20 mV s^−1^, and the potential window ranged from 0 to 0.9 V. After electrodeposition, the titanium mesh coated with polymers was immersed in distilled water to remove the soluble monomers or oligomers and finally air-dried at 70 °C. The mass of active material deposited on the titanium mesh (around 1 mg) was controlled by adjusting the CV cycles and measured on a precise analytical balance. Also, pure PANI was electrosynthesized in the absence of FeCl_2_ according to the abovementioned process.

### 2.2. Preparation of H_2_SO_4_/Fe^2+/3+^/Poly(Vinyl Alcohol) (PVA) Gel Electrolyte

H_2_SO_4_/PVA was obtained by mixing and stirring 5 g of PVA and 50 mL of 1 M H_2_SO_4_ together until it became stable and clear at 90 °C [45]. Subsequently, a certain amount of FeSO_4_·7H_2_O and Fe_2_(SO_4_)_3_ was added and the resultant mixture was stirred until it dissolved. Finally, the H_2_SO_4_/Fe^2+/3+^/PVA gel electrolyte was cooled down to ambient temperature. H_2_SO_4_/PVA gel electrolyte was also prepared without adding the ferric and ferrous salts.

### 2.3. Characterization

XRD was performed using an X-ray diffractometer (UItima IV) (Rigaku Corporation, The Woodlands, TX, USA) equipped with Co-Kα radiation, with the scanning angle from 3° to 60° at a rate of 10° min^−1^. FTIR transmission spectra were taken on an FTIR spectrometer (iS50, EQUINOX 55). The morphology of the samples was observed on a field emission scanning electron microscope (FESEM, S3700N).

### 2.4. Electrochemical Measurements

The CV, galvanostatic charge–discharge (GCD), and electrochemical impedance spectroscopy (EIS) were tested and recorded in either a two- or three-electrode system on a CHI 760e electrochemical analyzer (CH Instruments, Inc., Austin, State of Texas, USA). The electrolytes in the two- and three-electrode systems were 1 M H_2_SO_4_ and 1 M H_2_SO_4_/Fe^2+/3+^ redox-active electrolytes with different concentrations of Fe^2+/3+^ (0.2, 0.5, and 0.8 M), respectively. In the three-electrode system, an SCE, a platinum net, and polymer-coated titanium mesh were used as the reference electrode, counter electrode, and working electrode, respectively. For the solid-state SCs, the devices were assembled using polymer-coated titanium mesh as the electrodes and a H_2_SO_4_/Fe^2+/3+^/PVA gel electrolyte as both separator and electrolyte [45]. EIS measurements were performed in the frequency range of 100 kHz to 10 mHz at an AC amplitude of 5 mV.

The total capacitance of the SC (C_total_, F g^−1^), the specific capacitance of a single electrode (C_s_, F g^−1^), the energy density (E, W h Kg^−1^), and the power density (P, W Kg^−1^) were calculated according to these equations:

(1) $$C_{total}=\frac{I\times \Delta t}{V\times m}$$

(2) $$C_{s}=4\times C_{total}$$

(3) $$E=\frac{I\times \int \mathrm{Vd}t}{m}$$

(4) $$P=\frac{3600E}{\Delta t}$$

where *I* is the charge/discharge current (A), Δt is the discharge time (s), V is the potential drop in the discharge progress, ∫Vdt is the galvanostatic discharge current area, and m denotes the total mass of the PANI electrodeposited for both electrodes [42,43,46].

## 3. Results

**Figure 1:**
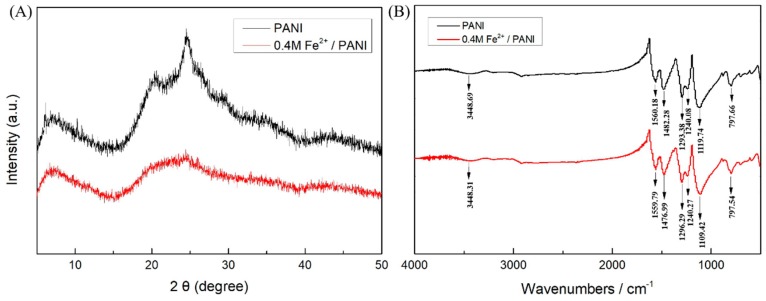
XRD pattern (**A**) and FTIR spectrum (**B**) of polyaniline (PANI) and 0.4 M Fe^2+^/PANI electrode material.

Fe^3+^-doped polyaniline has previously been prepared by an electrochemical method [21,47]. However, it was found that the electroplating electrolyte containing Fe^3+^ and aniline were very unstable and PANI precipitate occurred in tens of minutes, possibly due to the chemical reaction between Fe^3+^ and the monomer, as FeCl_3_ was often used as an oxidant reagent for the chemical polymerization of PANI. So here, Fe^2+^ was utilized in the electrolyte for the electrodeposition of PANI. The structures of the electropolymerized samples were studied by XRD and FTIR, respectively. In the XRD pattern (Figure 1A), three evident peaks were observed at 2θ = 7.1°, 20.5°, and 24.6°. The peak at 2θ = 7.1° confirmed the short-range ordered configuration of the polymer chains [42] and the others indicated the periodic structures parallel and vertical to the polymer chain in PANI, implying lower crystallinity and a conductive emeraldine salt structure [20,23]. Compared with PANI, the 0.4 M Fe^2+^/PANI electrode only showed two evident broad peaks at 2θ = 7.1° and 24.6°, and the weaker peaks indicated its lower crystallinity compared with PANI [47].

The characteristic peaks of PANI in Figure 1B were observed as follows: two bands located at 1560.18 and 1482.28 cm^−1^ were assigned to the stretching vibration of N=Q=N and N–B–N, where Q and B represent the quinoid and the benzenoid units, respectively. The bands located at 1293.38 and 1240.08 cm^−1^ were attributed to C–N and C=N stretching modes, respectively, while the band at 1119.74 cm^−1^ was related to the in-plane bending vibrations and the band at 797.66 cm^−1^ was due to the C–H bonds’ in-plane bending vibration in the 1,4-disubstituted aromatic ring [20,31,42,48]. It was noted that the peaks at 1560.18, 1482.28, and 1119.74 cm^−1^ displayed obvious red shifts after the modification with Fe^2+^ and appeared at 1559.79, 1476.99, and 1109.42 cm^−1^, respectively. The red shift implied the conversion of a quinone-like ring to a benzene ring by a proton-induced spin mismatch mechanism [49] and the decreasing charge delocalization in the main chain of PANI backbone, and the comprehensive effect of the protonation and pseudoprotonation processes had a decisive influence on the final chemical state of the PANI [31,50]. The XRD and FTIR spectrum indicated that PANI and Fe^2+^-modified PANI having an emeraldine chemical structure were prepared successfully and the PANI prepared in the presence of Fe^2+^ displayed lower crystallinity and charge delocalization on the PANI backbone.

**Figure 2:**
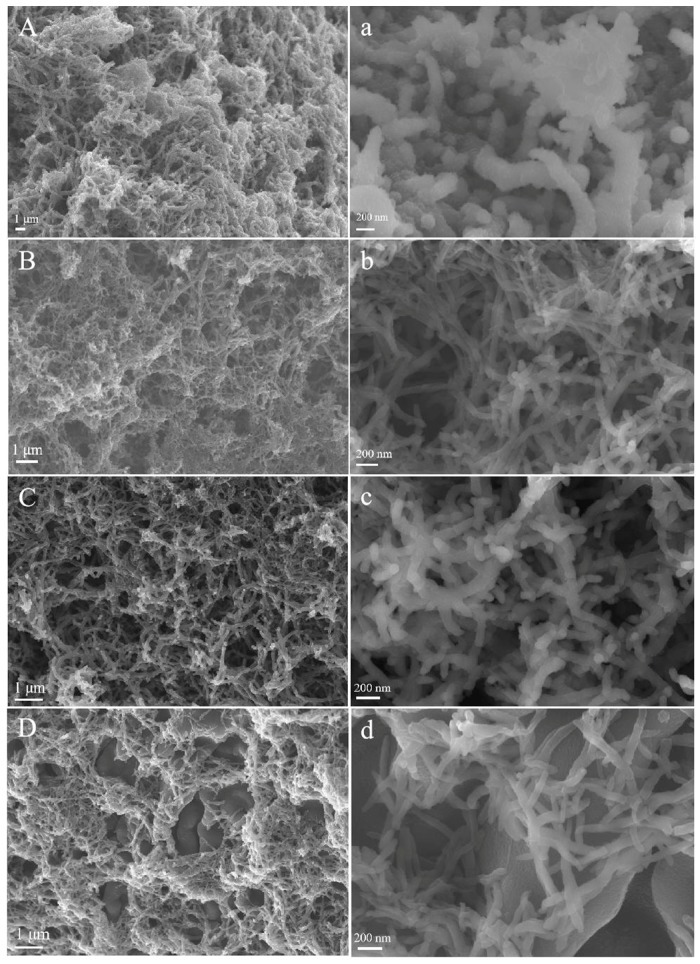
Field emission scanning electron microscopy (FESEM) images of PANI (**A**,**a**), 0.2 M Fe^2+^/PANI (**B**,**b**), 0.4 M Fe^2+^/PANI (**C**,**c**), and 0.8 M Fe^2+^/PANI (**D**,**d**) electrode materials.

The morphology of PANI and the other electrode materials were characterized by FESEM, as shown in Figure 2. All of the products showed a coral-like morphology which was similar to branched PANI nanofibers synthesized by chemical oxidation polymerization and electropolymerization with FeCl_3_ as an oxidizer, although the diameter of most of the Fe^2+^/PANI nanofibers here (~70 nm) was much larger than that of branched nanofibers (~40 nm) produced in previous works [51,52]. Obviously, the presence of metal cations during the electropolymerization of PANI affected its microstructure. Compared with the Fe^2+^/PANI with a clean fiber surface and a fiber diameter of around 70 nm (Figure 2b–d), the PANI obtained in the absence of Fe^2+^ displayed a much rougher fiber surface and a larger fiber diameter of 150–400 nm (Figure 2a). Interestingly, the 0.4 M Fe^2+^/PANI fibers seemed to be more branched than both the 0.2 M Fe^2+^/PANI and 0.8 M Fe^2+^/PANI nanofibers, which was beneficial for their higher electrochemical activity. According to their EDS analysis (Table S1), the Fe content in 0.4 M Fe^2+^/PANI was 3.03%. Based on the results of EDS and FTIR, the Fe^2+^ in the PANI may have attached to nitrogen atoms, which acted as the contact site in the polymer backbone, leading to the formation of branch-like PANI [53]. Unlike the PANI doped with other transition metallic ions (Ni^2+^, Cu^2+^, or Fe^3+^) [19,21,24], due to the coexistence of Fe^2+^ and aniline in the electrolyte, an excessive electrochemical reaction of Fe^2+^ occurred during the electropolymerization of PANI and had some influence on the electrochemical synthesis process, thus affecting the morphology of the products. As the nanostructure of the electrode materials has great positive effects on their electrode properties [54], the resultant ferrous-ion-modified PANI branched nanofibers were expected to exhibit improved electrochemical capacitive performance.

**Figure 3:**
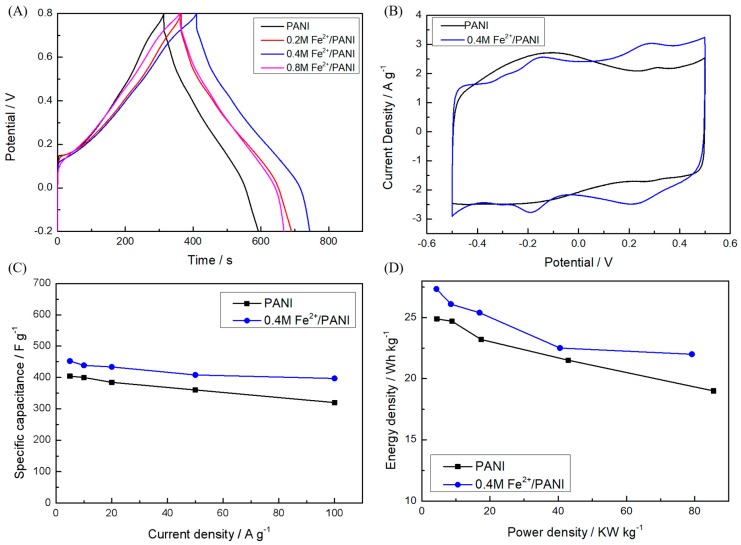
(**A**) Galvanostatic charge–discharge (GCD) curves of PANI and PANI doped with Fe^2+^ (0.2, 0.4, and 0.8 M) in a three-electrode system in 1 M H_2_SO_4_ at a current density of 1 A g^−1^, respectively. Electrochemical performance of symmetric PANI and 0.4 M Fe^2+^/PANI supercapacitors (SCs) with 1 M H_2_SO_4_ electrolyte: (**B**) cyclic voltammetry (CV) curves at a scan rate of 20 mV s^−1^, (**C**) specific capacitances at different current densities, and (**D**) Ragone plots.

The electrochemical performance of the electropolymerized PANI materials was first tested in the three-electrode system in 1 M H_2_SO_4_. In Figure 3A, the discharge times of all three Fe^2+^/PANI electrodes at 1 A g^−1^ were longer than pure PANI, indicating they had a higher C_s_ than that of PANI. The corresponding C_s_ was 328 F g^−1^ for 0.2 M Fe^2+^/PANI, 335 F g^−1^ for 0.4 M Fe^2+^/PANI, and 304 F g^−1^ for 0.8 M Fe^2+^/PANI, all of which were larger than the 298.8 F g^−1^ of pure PANI. Obviously, the polymer deposited in the presence of 0.4 M Fe^2+^ exhibited the highest C_s_ among these electrodes. Therefore, 0.4 M Fe^2+^/PANI was used to assemble SCs in the following work.

The PANI or Fe^2+^/PANI electrodes were used to assemble the symmetric SCs with the 1 M H_2_SO_4_/PVA gel electrolyte. The CV curves of 0.4 M Fe^2+^/PANI and PANI (Figure 3B) were similar and both of them showed a rectangular form with a little deviation. There was a pair of evident redox peaks in the curves between 0.2 and 0.4 V for both pure PANI and 0.4 M Fe^2+^/PANI, which was attributed to the conversion between the semiconducting (leucoemeraldine form) and conducting (polaronic emeraldine form) states. Another evident pair of redox peaks occurred between −0.2 and −0.1 V for the 0.4 M Fe^2+^/PANI electrode, which was close to that of a similar work on symmetric PANI SCs with a 0.5 M H_2_SO_4_ electrolyte containing an Fe^2+^/Fe^3+^ additive [42]. However, its peak difference (~70 mv) here was clearly much less than that which was contributed by the Fe^2+^/Fe^3+^ additive in the electrolyte [42], which could have been due to the active transition metal ion doping inside the PANI electrode materials in our work rather than dissolving in the electrolyte. These results indicate the successful doping of Fe^2+^ in PANI and the corresponding change of its electrochemical behavior. According to the GCD tests at a current density of 5 A g^−1^ (Figure 3C), the C_s_ of the 0.4 M Fe^2+^/PANI electrode was 452 F g^−1^, which was much higher than that of pure PANI (404 F g^−1^). Moreover, a better rate capability of the materials could be achieved when the electrodeposition of PANI was carried out in the presence of Fe^2+^. For pure PANI, 79.21% of C_s_ could be preserved when the GCD current was increased from 5 to 100 A g^−^^1^, while 87.79% of C_s_ still remained for 0.4 M Fe^2+^/PANI, as shown in Figure 3C. Furthermore, the results of the cyclic stability tests also demonstrated the positive effect of the Fe^2+^ modification of PANI. As shown in Figure S1, after 1000 cycles at 20 A g^−^^1^, the C_s_ of 0.4 M Fe^2+^/PANI displayed only a slight drop of 0.41%, which was much less than that of PANI (12.5%). The positive effect of Fe^2+^ doping may originate from the additional redox process and extra active sites relative to the metal ions in PANI, which stabilized and enhanced the performance of PANI.

**Figure 4:**
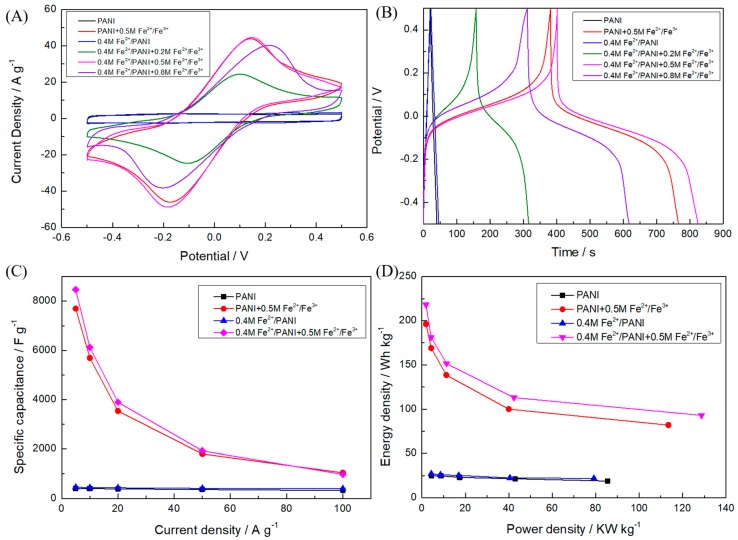
Electrochemical performance of symmetric PANI and 0.4 M Fe^2+^/PANI SCs with 1 M H_2_SO_4_ + Fe^2+^/Fe^3+^ (0, 0.2, 0.5, and 0.8 M) electrolytes: (**A**) CV curves at a scan rate of 20 mv s^−1^, (**B**) GCD curves at a current density of 5 A g^−1^, (**C**) specific capacitances at different current densities, and (**D**) Ragone plots.

To improve the performance of the capacitors further, Fe^2+^/Fe^3+^ redox couples were introduced into the electrolyte in the SCs. In contrast to the nearly rectangular CV curve in SCs with only the H_2_SO_4_ electrolyte (Figure 3B), it can be seen that there was one pair of strong redox peaks with good symmetry in the CV curves of both PANI and 0.4 M Fe^2+^/PANI electrodes in the redox-active electrolyte (Figure 4A), which clearly shows the contribution of the redox couple in the electrolyte [44]. The peak potential difference (|Epc-Epa|) increased with the increase of the concentration of the redox additive in the electrolyte. The redox peak current first increased as the concentration of Fe^2+^/Fe^3+^ increased from 0.2 to 0.5 M and then noticeably decreased after reaching 0.8 M. The results indicate that a proper ion concentration helps to enhance the electrochemical performance of SCs, although an excessive metal ion concentration may reduce water hydration, thus decreasing ion activity and mobility [44]. Also, the redox peak current of 0.4 M Fe^2+^/PANI increased slightly relative to that of PANI, which could have been due to the limited contribution of the metal ions doped in the electrode material to the whole electrochemical process in the redox-active electrolyte. The discharge time of SCs increased when the concentration of Fe^2+^/Fe^3+^ in the electrolyte increased from 0 to 0.5 M and then decreased when the concentration of the redox couple increased to 0.8 M, as shown in the GCD curves obtained at 5 A g^−^^1^ (Figure 4B). Correspondingly, the calculated C_s_ of the 0.4 M Fe^2+^/PANI electrodes was enhanced from 452 F g^−^^1^ in the H_2_SO_4_ electrolyte to 8468 F g^−^^1^in H_2_SO_4_ + 0.5 M Fe^2+^/Fe^3+^. The increased C_s_ could have been due to the additional electrochemical reaction of the Fe^2+^/Fe^3+^ additive in the electrolyte on the active sites of the electrodes [42]. By adjusting the concentration of the active additive in the SCs and performing the relative GCD tests, its optimum concentration could be obtained. Also, the C_s_ values of the 0.4 M Fe^2+^/PANI electrodes in the electrolyte with the addition of 0.2 and 0.8 M Fe^2+^/Fe^3+^ was 3150 and 6160 F g^−^^1^, respectively, indicating the optimum Fe^2+^/Fe^3+^ in the electrolyte was 0.5 M. These C_s_ values were much larger than others which have been reported in the literature (Table 1), and this could have been due to the advantage of modifying both the electrode material and the electrolyte with the redox-active additive in this work.

**Figure 5:**
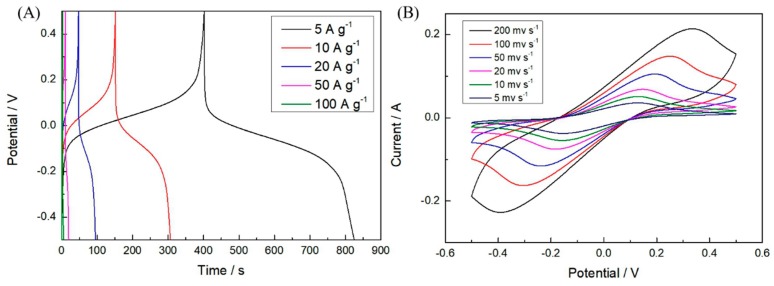
Electrochemical performance of symmetric 0.4 M Fe^2+^/PANI SCs in 1 M H_2_SO_4_ + 0.5 M Fe^2+^/Fe^3+^ electrolyte: (**A**) GCD curves at different current densities and (**B**) CV curves at different scan rates.

To study the rate capability of the SCs with the redox-active additive in the electrolyte, their GCD measurements were performed at different current densities. For both PANI and 0.4 M Fe^2+^/PANI electrode materials, a sharp decline of C_s_ with the increasing GCD current density could be observed, as shown in Figure 4C. This can be ascribed to the insufficiency of the redox reaction of Fe^2+^/Fe^3+^ at high current densities. The C_s_ of the 0.4 M Fe^2+^/PANI in 1 M H_2_SO_4_ + 0.5 M Fe^2+^/Fe^3+^ electrolyte gradually decreased from 8468 to 6120, 3896, 1924, and 972 F g^−^^1^ as the current increased from 5 to 10, 20, 50, and 100 A g^−^^1^, respectively, which was calculated according to the GCD curves in Figure 5A. The C_s_ retention of 11.47% at 100 A g^−1^ could have been due to the inadequate redox reaction of Fe^2+^/Fe^3+^, the concentration polarization, and the reduced accessible area during the charge–discharge process with such a high current density. However, this value was still much larger than PANI in the normal H_2_SO_4_ electrolyte. To the best of our knowledge, this is the first report of such a remarkable C_s_ at high current densities for PANI-based symmetric SCs. Similarly, the CV curves at higher scan rates showed an increased peak potential difference (Figure 5B).

The energy density and power density of energy storage devices are very important for their practical application. As can be seen in Figure 4D, the 0.4 M Fe^2+^/PANI symmetric SC with 1 M H_2_SO_4_ + 0.5 M Fe^2+^/Fe^3+^ electrolyte processed an energy density as high as 218.1 Wh kg^−1^ at a power density of 1854.4 W kg^−1^, which was not only better than that of the other samples, such as PANI in a 1 M H_2_SO_4_ electrolyte (24.9 Wh kg^−1^ at 4437.6 W kg^−1^), 0.4 M Fe^2+^/PANI in a 1 M H_2_SO_4_ electrolyte (27.35 Wh kg^−1^ at 4318.4 W kg^−1^) (Figure 3D), and PANI in a 1 M H_2_SO_4_ + 0.5 M Fe^2+^/Fe^3+^ electrolyte (196 Wh kg^−1^ at 1834.6 W kg^−1^) (Figure 4D), but also higher than those of other reports on PANI-based electrode materials tested in a H_2_SO_4_ + Fe^2+^/Fe^3+^ electrolyte, including PANI (22.1 Wh kg^−1^ at 774.0 W kg^−1^) [42] and PANI/CNT (22.9 Wh kg^−1^ at 700.1 W kg^−1^) [43].

**Figure 6:**
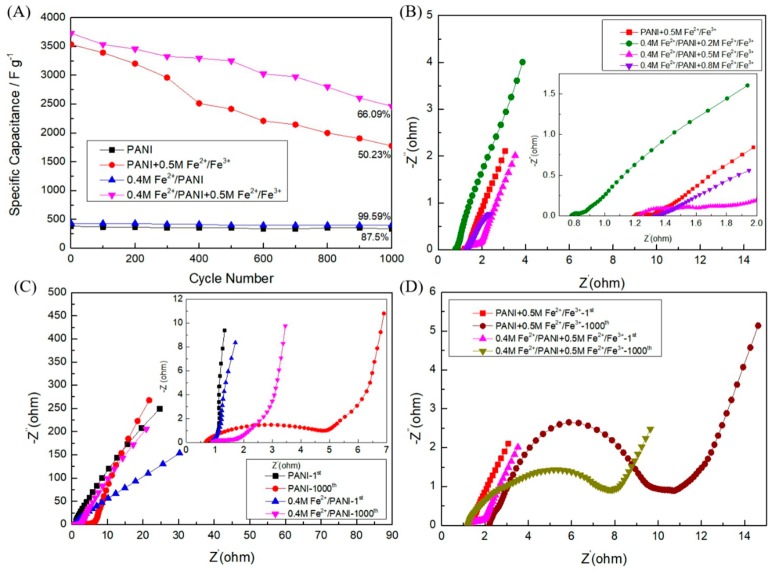
Cycling performance of symmetric PANI and 0.4 M Fe^2+^/PANI SCs with 1 M H_2_SO_4_ + Fe^2+^/Fe^3+^ (0 and 0.5 M) electrolytes: (**A**) capacitance retention at 20 A g^−1^ for 1000 cycles; Nyquist plots of (**B**) symmetric PANI and 0.4 M Fe^2+^/PANI SCs with 1 M H_2_SO_4_ + Fe^2+^/Fe^3+^ (0, 0.2, 0.5, and 0.8 M) electrolytes (inset: the close-up view of the high-frequency region); (**C**) symmetric PANI and 0.4 M Fe^2+^/PANI SCs with a 1 M H_2_SO_4_ electrolyte (inset: the close-up view of the high-frequency region); and (**D**) symmetric PANI and 0.4 M Fe^2+^/PANI SCs with a 1 M H_2_SO_4_ + 0.5 M Fe^2+^/Fe^3+^electrolyte before and after 1000 cycles.

To further study the effect of both modifying the electrode materials and utilizing the redox-active gel electrolyte on the performance of SCs, the cycle stability of the assembled SCs was tested for 1000 charge–discharge cycles at 20 A g^−1^. As shown in Figure 6A, after testing for 1000 cycles in a 1 M H_2_SO_4_ + 0.5 M Fe^2+^/Fe^3+^ electrolyte, 0.4 M Fe^2+^/PANI and PANI electrode materials showed a C_s_ retention of 66.09% and 50.23%, respectively. Meanwhile, 0.4 M Fe^2+^/PANI showed better C_s_ retention (99.59%) than that of PANI (87.5%) in 1 M H_2_SO_4_ (Figure S1), indicating that by modifying PANI with Fe^2+^, the cyclic stability of the electrode can be also enhanced greatly in the redox-active electrolyte. Similar cyclic stability improvements have been reported in the case of PANI films doped with Fe^3+^ in an H_2_SO_4_ electrolyte [21] and could be attributed to the synergistic effect of PANI and metal ion dopants and the ability to better adjust the volume change during the electrochemical process [47], originating from the electrochemical redox reaction of both PANI and transition metal ions in the electrode composite materials, as well as their thinner and highly branched fiber structure (Figure 2c), which provide more redox reaction active sites and room to accommodate the volume expansion and shrinkage of PANI during charge–discharge of SCs. It is also noted that the cyclic stability of PANI and 0.4 M Fe^2+^/PANI in the H_2_SO_4_ + Fe^2+^/Fe^3+^ electrolyte was inferior to that in the H_2_SO_4_ electrolyte, which could have been due to the extensive redox reaction caused by the redox additive in the electrolyte and the destruction of the polymer chain by the additional electrochemical process of the Fe^2+^/Fe^3+^ redox couple on the electrode materials [56]. The electrochemical behavior of all-solid-state flexible SCs were also characterized during the deformation of the SCs. In Figure S2, the GCD curves of 0.4 M Fe^2+^/PANI SCs with a 1 M H_2_SO_4_ + 0.5 M Fe^2+^/Fe^3+^ electrolyte tested under bending and twisting changed little, indicating the good capacitive stability of the assembled flexible PANI-based SCs with high performance at different deformations.

Nyquist plots of PANI SCs and 0.4 M Fe^2+^/PANI SCs with different concentrations of Fe^2+^/Fe^3+^ in a 1 M H_2_SO_4_ electrolyte are shown in Figure 6B. In the high-frequency region, the initial nonzero intercept of the semicircle on the real axis represents the combined series resistance of the electrolyte, electrode, current collector, and contact resistance of the active material/current collector (Rs), and the minimum value of Rs means a greater conducting nature of the composite electrodes in the electrochemical system [20,57]. Another significant feature of the plots is the nearly vertical line to the abscissa axis in the low-frequency region, which corresponds to the resistance of transport and the diffusion process in the electrolyte [58,59,60]. The Rs value of 0.4 M Fe^2+^/PANI in the 1 M H_2_SO_4_ electrolyte was 0.872 Ω, which was much less than that in the 1 M H_2_SO_4_ + 0.5 M Fe^2+^/Fe^3+^ electrolyte (1.218 Ω). Moreover, as Fe^2+^/Fe^3+^ in the electrolyte increased from 0.2 to 0.8 M, the Rs value increased significantly from 0.782 to 1.329 Ω, indicating the evident resistance contribution of the high concentration of Fe^2+^/Fe^3+^ in the electrolyte to the combined series resistance in the SCs. Also, both the Rs of PANI in the 1 M H_2_SO_4_ (Figure 6C) and the 1 M H_2_SO_4_ + 0.5 M Fe^2+^/Fe^3+^ (Figure 6B) electrolyte was less than that of 0.4 M Fe^2+^/PANI, which can be attributed to the amorphous configuration of PANI with lower charge delocalization and a thinner polymer nanofiber structure formed in the presence of Fe^2+^. The pseudocapacitance contributed by both Fe^2+^/PANI electrodes and the Fe^2+^/Fe^3+^ redox additive in the electrolyte resulted in the oblique line deviating from a perfect vertical line in the low-frequency region, the diffusion-controlled doping/undoping of PANI, and the Fe^2+^/Fe^3+^ redox reaction leading to the Warburg behaviors as shown in the plots [43,61].

In addition, the Nyquist plots of PANI and 0.4 M Fe^2+^/PANI with Fe^2+^/Fe^3+^ (0 and 0.5 M) in the electrolyte were also recorded before and after 1000 GCD cycles. Interestingly, before the first cycle, the Rs value of 0.4 M Fe^2+^/PANI in the 1 M H_2_SO_4_ electrolyte was 1.202 Ω, which was bigger than that of PANI in the same electrolyte (0.824 Ω), indicating that the Fe^2+^-modified PANI electrodes were less conductive. This is consistent with the aforementioned XRD and FTIR analyses. In Figure 6C,D, the diameter of the semicircle at the high-frequency region represents the charge-transfer resistance (Rct), which is related to the ion diffusion between the electrode and electrolyte interface [62,63]. After 1000 cycles, for both PANI and 0.4 M Fe^2+^/PANI in 1 M H_2_SO_4_ + Fe^2+^/Fe^3+^ (0 or 0.5 M) electrolytes, the Rct became larger than that before the first cycle. This implies the volume expansion of the polymer and/or the decrease of the active sites after 1000 cycles. Especially with the incorporation of Fe^2+^/Fe^3+^ in the electrolyte, the additional redox reaction of iron ions at the electrode/electrolyte interface may have caused higher charge-transfer resistance and harder ion diffusion in the SCs.

## 4. Conclusions

In summary, Fe^2+^-modified PANI materials were prepared and optimized by electropolymerization. The effects of Fe^2+^ modification on the physical and chemical properties of electropolymerized PANI were studied. The PANI prepared in the presence of Fe^2+^ displayed a thinner and more branched fiber structure than that synthesized in the absence of Fe^2+^. The C_s_ and capacitance retention of 0.4 M Fe^2+^/PANI electrode materials in the 1 M H_2_SO_4_/PVA electrolyte was 452 F g^−1^ and 99.59% after 1000 cycles at 5 A g^−1^, respectively, both of which were much higher than that of its counterpart prepared without the presence of Fe^2+^. An Fe^2+^/Fe^3+^ redox couple additive was also introduced into the electrolyte, and its concentration in the electrolyte was optimized to be 0.5 M. As a result, 0.4 M Fe^2+^/PANI electrodes tested in the 1 M H_2_SO_4_ + 0.5 M Fe^2+^/Fe^3+^ gel electrolyte exhibited a remarkable C_s_ of 8468 F g^−1^ at 5 A g^−1^. Moreover, the resultant SCs exhibited enhanced energy density, which was nearly 9 times that of SCs with PANI electrodes without the Fe^2+^ modification and the Fe^2+^/Fe^3+^ additive in the electrolyte and was much higher than that of other reports on PANI symmetric SCs with an H_2_SO_4_ + Fe^2+^/Fe^3+^ electrolyte. This work demonstrated an effective strategy to fabricate SCs with excellent electrochemical performance through the modification of conductive polymer electrodes with transition metal ions, as well as by adding a redox-active additive in the electrolyte.

## Figures and Tables

**Table 1:** Comparison of specific capacitance (C_s_) values of PANI-based electrode materials.

Material Fabrication Method		C_s_/F·g^−1^	Electrolyte	Citation
PANI	Chemical oxidative polymerization	1062/2 A g^−1^	1 M H_2_SO_4_ + 0.8 M Fe^3+/2+^	[42]
PANI/SnO_2_	Chemical oxidative polymerization	1172/1 A g^−1^	1 M H_2_SO_4_ + 0.4 M Fe^3+/2+^	[44]
PANI/CNT	Electrodeposition	1128/5 A g^−1^	1 M H_2_SO_4_ + 0.02 M Fe^3+/2+^	[43]
Fe^3+^-Zn^2+^-PANI/GO	Electrodeposition	1140/10 A g^−1^	0.5 M Na_2_SO_4_	[47]
PANI/RGO	Chemical oxidative polymerization	565/0.1 A g^−1^	6 M KOH	[55]
Fe^3+^/PANI	Electrodeposition	602/3 mA cm^−2^	0.5 M H_2_SO_4_	[21]
Fe^2+^/PANI	Electrodeposition	8468/5 A g^−1^	1 M H2SO4 + 0.5 M Fe^3+/2+^	This work

## Supplementary Materials

The following are available online at https://www.mdpi.com/2073-4360/11/8/1357/s1, Table S1: EDS of PANI and 0.4 M Fe ^2+^/PANI (atomic concentration), Figure S1: Cycling performance of symmetric PANI and 0.4 M Fe^2+^/PANI SCs in 1 M H_2_SO_4_ electrolytes: capacitance retention at 20 A g^−1^ for 1000 cycles, Figure S2: GCD curves of all-solid-state device under normal, bending, and flexibility conditions.
