# Supplementary material for: Redox-Active Gel Electrolyte Combined with Branched Polyaniline Nanofibers Doped with Ferrous Ions for Ultra-High-Performance Flexible Supercapacitors
Source: Polymers (Basel). 2019 Aug 16;11(8):1357. doi: 10.3390/polym11081357 (PMC6722530; doi:10.3390/polym11081357)
Supplement: Supplementary file 1 [file polymers-11-01357-s001.pdf]

Supporting information

Table S1. EDX of PANI and 0.4M Fe^{2+}/PANI (atomic concentration)

Sample	C	N	O	Cl	Fe
PANI	71.48	20.53	3.07	4.92	-
0.4M Fe^{2+}/PANI	63.12	18.91	5.25	9.69	3.03

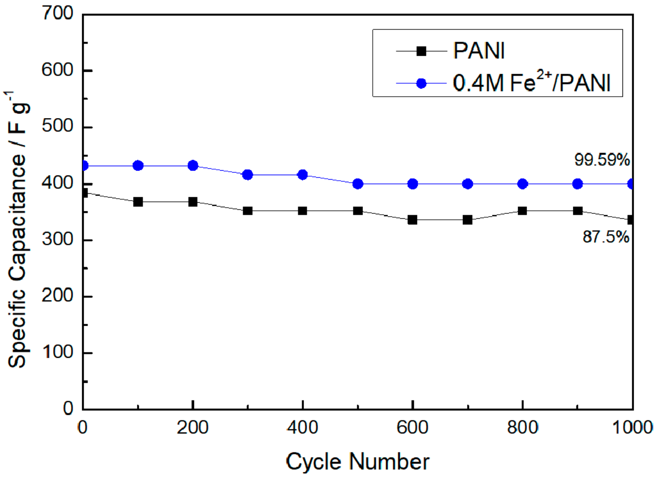

Figure S1. Cycling performance of symmetric PANI and 0.4M Fe^{2+}/PANI SCs in 1M H_2SO_4 electrolytes: capacitance retention at 20 A g^{-1} for 1000 cycles.

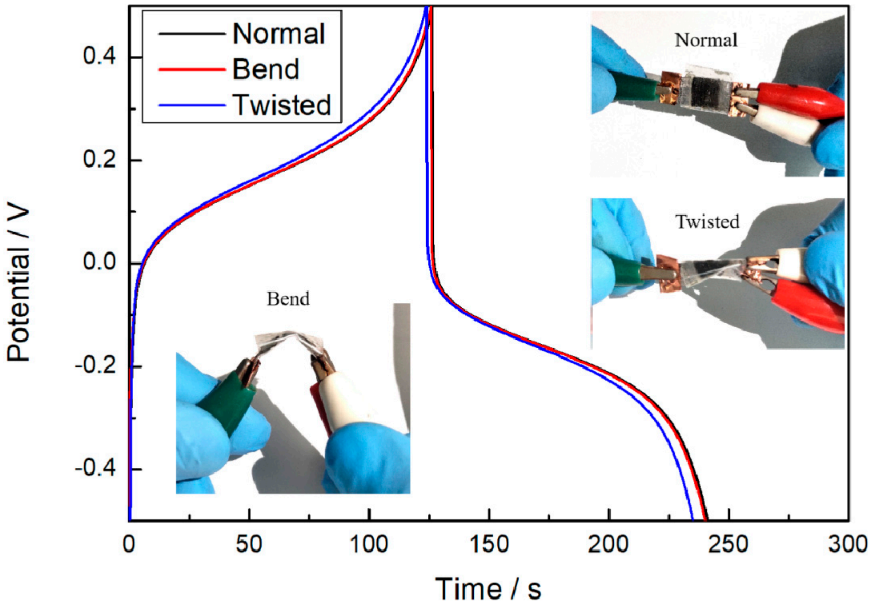

Figure S2. GCD curves of all-solid-state SC device under different deformation conditions.
